# The Role of Solvents in Lithography-Based Ceramic Manufacturing of Lithium Disilicate

**DOI:** 10.3390/ma14041045

**Published:** 2021-02-23

**Authors:** Malte Hartmann, Markus Pfaffinger, Jürgen Stampfl

**Affiliations:** Institute of Materials Science and Technology, Faculty of Mechanical and Industrial Engineering, Technische Universität Wien, Getreidemarkt 9, 1060 Vienna, Austria; markus.pfaffinger@cubicure.com (M.P.); juergen.stampfl@tuwien.ac.at (J.S.)

**Keywords:** refractive index, debinding, glass ceramics, digital dentistry, additive manufacturing, vat photo-polymerization

## Abstract

Digital dentistry is increasingly replacing conventional methods of manually producing dental restorations. With regards to computer-aided manufacturing (CAM), milling is state of the art. Additive manufacturing (AM), as a complementary approach, has also found its way into dental practices and laboratories. Vat photo-polymerization is gaining increasing attention, because it enables the production of full ceramic restorations with high precision. One of the two predominantly used ceramic materials for these applications is lithium disilicate, Li_2_Si_2_O_5_. This glass ceramic exhibits a substantial fracture toughness, although possesses much lower bending strength, than the other predominantly used ceramic material, zirconia. Additionally, it shows a much more natural optical appearance, due to its inherent translucency, and therefore is considered for anterior tooth restorations. In this work, an optimized formulation for photo-reactive lithium disilicate suspensions, to be processed by vat photo-polymerization, is presented. Following the fundamental theoretical considerations regarding this processing technique, a variety of solvents was used to adjust the main properties of the suspension. It is shown that this solvent approach is a useful tool to effectively optimize a suspension with regards to refractive index, rheology, and debinding behavior. Additionally, by examining the effect of the absorber, the exposure time could be reduced by a factor of ten.

## 1. Introduction

In dentistry, the development of computer-aided design/computer-aided manufacturing (CAD/CAM) systems in the 1980s brought a significant change to the field [1]. Continuous automatization and computerization finally led to the formation of a so-called digital dentistry, where the whole workflow—from assessment of the dentition situation by modern scan methods, via modelling of the restoration by adapted software, to the automated production of the restoration by CAM—is completed digitally. All necessary data is recorded virtually, so that, ideally, the manual preparation of impressions or models can be discarded.

The CAM method that has been developed primarily for this case is milling. This technique is now state of the art. In recent years, additive manufacturing (AM) has gained increasing attention as a complementary approach and, for certain cases, has found its way into dental laboratories and practices [2]. To enable a successful restoration, like a crown or bridge, high precision and good surface quality are necessary. Vat photo-polymerization is an AM method that excels in these categories. By combining this technology with the colloidal processing route, the production of full ceramic parts is possible [3].

This work focuses on lithography-based ceramic manufacturing (LCM) with regards to the technology and lithium disilicate with regards to the material. Lithium disilicate is one of the materials used predominantly for full ceramic restorations (the other one being zirconia) [4]. In LCM, suspensions or pastes are used, whose formulation can be rather complex. Changing one constituent or the fractions of the composition can easily result in a negative influence on other properties of the formulation. Here an approach was chosen that includes a change of the solvent composition. The aim was to change certain properties of the suspension, such as refractive index, rheology, and debinding behavior, without interfering too much with the polymerization mechanism. Table 1 lists typical components for such a suspension. In the following, these components and considerations of importance regarding their selection are discussed.

The starting point for the formulation is the filler. Amount of solid loading, particle size, particle size distribution, shape of the powder particles, and chemical constitution are factors that must be evaluated when developing a formulation. Amount of solid loading, particle size, and particle shape mainly determine the rheological behavior of the suspension. The viscosity, for example, increases with increasing solid loading and deviation from spherical particle shape [5,6].

The chemical constitution of the powder is pivotal for the strategy of stabilizing the suspension against agglomeration and sedimentation. Colloidal suspensions are divided into lyophilic and lyophobic colloids. In lyophilic colloids, the solvent has a strong affinity to the dispersed particles. In this case, the liquid is strongly adsorbed on the surface, and the system is stable by itself, because the free Gibbs energy is decreased during the process of dispersing. On the other hand, lyophobic colloids show no affinity between liquid and particle surface. The free Gibbs energy increases during the process of dispersing and attractive forces between the particles are active, which is the reason for quick agglomeration. In most cases, ceramic suspensions are lyophobic colloids [7]. Therefore, it is useful to acquire knowledge about what kind of attractive forces appear during dispersing and how they can be overcome by purposefully introducing repulsive forces to create suspensions with the desired stability.

A meaningful tool with regards to these considerations is the dispersing agent. By binding to the particle surface, it allows one to specifically introduce repulsive forces. In general, the following strategies are used for this purpose: electrostatic stabilization, steric stabilization, or electrosteric stabilization. In the case of suspensions in organic liquids, as they come into use in, for example, slip casting or tape casting, mostly steric stabilization is applied, where unloaded polymer chains are adsorbed on the particle surface [8]. Stable suspensions have the advantage that, in LCM, they enable higher homogeneity with regards to the solid loading of green bodies. In the case of unstable suspensions, over the course of the printing process, segregation and sedimentation take place, which leads to a different solid loading of the green bodies, depending on the height of the printed object [9].

Thixotropy is an effective instrument to prevent sedimentation and, at the same time, enables a good processability. It is defined as: “A time dependency of the flowing properties of non-Newtonian fluids, whose viscosity is decreased by continuous external influences and returns to starting viscosity only after the ceasing of load [10]”. It is important to stress that in addition to shear-thinning behavior, thixotropic materials exhibit a time dependency of the viscosity recovery (i.e., after a mechanical load has been removed for a certain time period, the viscosity returns to its initial state).

In LCM, this is extensively practical. As long as no shearing forces are applied to the suspension (e.g., during storage), the suspension is ideally highly viscous and stable. When shearing forces are applied by the doctor blade during coating, the structural strength of the suspension is decreased. This way, the suspension yields to the load and enables easy processing. After the coating process, thixotropy leads to an increase of structural strength and therefore to the stability of the suspension after a given time period. This way, introducing a thixotropic additive enables the stabilization during storage and processing of an otherwise unstable suspension [11].

Since the basic mechanism for layer generation in LCM is based on photo-polymerization, optical properties like the refractive index are especially relevant for this method. When the difference in refractive index between dispersing phase and dispersed phase is high, the penetration depth of light is low and the widening, due to scattering, is high. When the difference in refractive index is low, penetration depth is high and widening low. To enable a high precision in LCM, it is necessary to target a small difference in refractive index (refractive index matching). The penetration depth can afterwards be decreased by utilizing an appropriate concentration of absorber [12].

Absorbers are chemical substances, which, ideally, are inert with regards to the polymerization and, by absorbing photons, reduce the penetration depth of light into the material. In vat photo-polymerization, the penetration depth determines the z-resolution. This is significant, for example, in the case of overhanging features of the part to be built. As the currently built part is always surrounded by redundant photo-reactive suspension, overhangs will inevitably be filled with cured material if the penetration (cure depth) is considerably higher than the layer thickness. Therefore, the cure depth should be of the same magnitude as the layer height. By varying the concentration of absorber, the cure depth can be adjusted for a given exposure. Therefore, it is essential that an absorbing agent efficiently absorbs at the emission wavelength of the light source. If the substance absorbs in the visible wavelength spectrum, it is called a dye [13]. Using an absorber leads to a higher conversion of double bonds at the same cure depth [14]. Understanding this is essential for the additive manufacturing process. The cure depth can also be decreased by reducing the exposure, but layers generated in this manner possess only insufficient mechanical stability and easily rupture (e.g., when detached from the vat or, in the worst case, when only gelled).

Being another main constituent of typical photo-reactive suspensions, the solvent has several functions. Firstly, it dissolves the solid organic constituents (photo-initiator and absorber). Secondly, it reduces the viscosity of the suspension. Thirdly, it assists the debinding process. Thermal debinding is by far the most implemented method. In this case, the binder is removed by heating, typically at atmospheric pressure and in oxidizing or non-oxidizing atmosphere. The mechanisms and physico-chemical factors that are fundamental to this process have been examined intensively in the literature. The debinding is controlled by heat transfer into the green body and mass transport of volatile substances and waste products, through the filled and unfilled pores, to the outside of the green body. It is important to mention that ceramic particles can influence the degradation process of the pure polymer [15].

As mentioned, debinding often takes place in oxidizing atmosphere (in many cases ambient air). It was shown that the oxidizing atmosphere at first hinders the chemical degradation of poly(methyl methacrylate) at low temperatures (200–300 °C), but accelerates it at higher temperatures later. In any case, it is a core property of the oxidizing atmosphere to reduce the amount of residual carbon in ceramic bodies [16].

In general, the debinding process can be divided into three phases. In phase one, warming takes place until the softening of the binder starts (lower than 200 °C). The chemical degradation is limited in this phase, but volatile lower-molecular-weight species (e.g., solvents) must diffuse through binder-filled pores and convert into the gas phase. The rapid generation of these species and the presence of dissolved air or captured air bubbles can lead to defects in the ceramic body [17]. In phase two (200–300 °C), higher-molecular-weight products are quickly degraded, and the diffusion and degassing of the resulting degradation products takes place. If the softened/molten binder possesses enough mobility, a redistribution of binder can happen, and therefore a non-planar debinding front can appear. This can ease and accelerate the exit of volatile species. Lower-molecular-weight species, like plasticizers or waxes, can leave a concentration gradient behind [18]. In phase three (300–400 °C), the rest of the binder is slowly removed by degradation and degassing. This process is now alleviated by the high porosity of the brown body, but a residual amount of carbon is still present. Often, temperatures up to 600 °C or higher are necessary to significantly reduce this residual amount of carbon.

## 2. Materials and Methods

### 2.1. Materials

#### 2.1.1. Solvent

**Figure 1:**
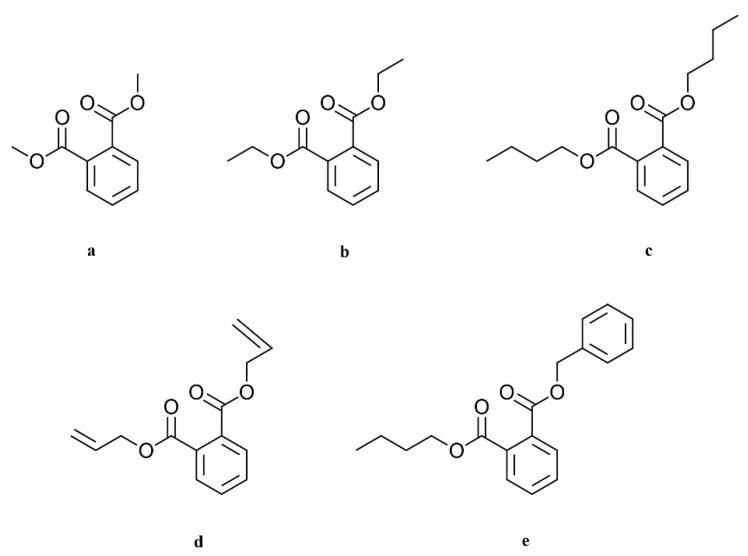
Chemical structures of the respective phthalates: (**a**) dimethyl phthalate, (**b**) diethyl phthalate, (**c**) dibutyl phthalate, (**d**) diallyl phthalate, (**e**) benzyl butyl phthalate.

Since preliminary experiments with phthalates showed promising results, this group of solvents was used for further investigation [9]. From the group of phthalates, dimethyl (DMP), diethyl (DEP), dibutyl (DBP), diallyl (DAP), and benzyl butyl phthalate (BBP) were used. All of them were obtained from Sigma-Aldrich (Merck KGaA, Darmstadt, Germany). The structural formulas of the phthalates are shown in Figure 1.

#### 2.1.2. Monomers

**Figure 2:**
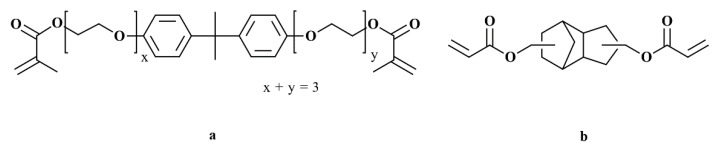
Chemical structures of (**a**) SR348C and (**b**) SR833S.

Two monomers under the denotations SR348C and SR833S were used exclusively. They were obtained from the company Sartomer (Arkema, Colombes, France). SR348C is a difunctional ethoxylated bisphenol-A dimethacrylate with a high viscosity (550–1700 mPa∙s at 25 °C). SR833S is a difunctional tricyclodecane dimethanole diacrylate with a low viscosity (120–160 mPa∙s at 25 °C). Both structural formulas are given in Figure 2.

#### 2.1.3. Dispersant

The dispersant is a commercially available substance called Solplus K500, produced by the company Lubrizol (Wickliffe, OH, USA). It is a brown, highly viscous, completely polymeric additive, which, according to the producer, is suitable for inorganic fillers in plasticizer suspensions. The chemical structure is not listed. The producer recommends an addition of 1–3% with regards to the mass of the filler.

#### 2.1.4. Photo-Initiator

**Figure 3:**
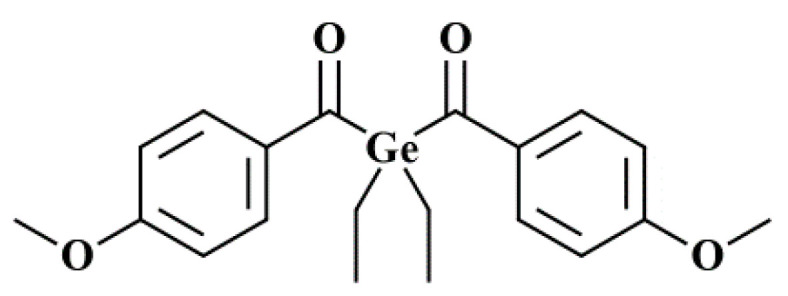
Chemical structure of the photo-initiator Ivocerin.

For this work, the photo-initiator Ivocerin was used exclusively. It was provided by our project partner Ivoclar Vivadent AG (Schaan, Liechtenstein). The chemical structure is shown in Figure 3. A specialty of this molecule is its considerable absorbance in the visible wavelength spectrum [19].

#### 2.1.5. Absorber

**Figure 4:**
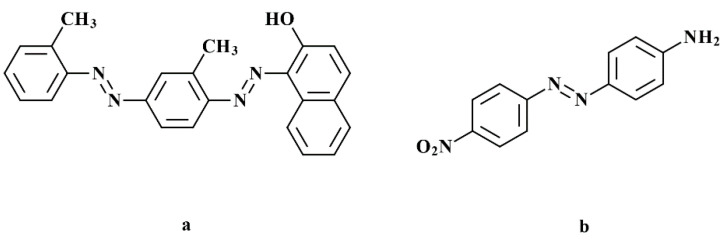
Chemical structures of (**a**) Sudan IV and (**b**) Disperse Orange 3.

The two azo-dyes Sudan IV and Disperse Orange 3 served as absorbers. They were obtained from Sigma-Aldrich (Merck KGaA, Darmstadt, Germany). The structural formulas are given in Figure 4.

#### 2.1.6. Filler

As filler, the glass precursor for lithium disilicate was used. After green part production, during sintering, the crystalline phase is generated. The glass precursor has the denotation PU GM e.max LT Transpa and was provided by our project partner Ivoclar Vivadent AG (Schaan, Liechtenstein). The chemical composition is stated in Table 2.

### 2.2. Methods

#### 2.2.1. Sample Preparation

Firstly, the respective organic materials were merged according to the composition given in Table 3. The glass powder was added in two steps, after each of which a mixing time of one minute at 2350 rpm was applied. A solid loading of 72.55% with regards to the mass was utilized. Afterwards, for complete dispersion and degassing, an additional mixing program was applied, which is given in Table 4. The machine software allows the combination of several steps into one program. For the program shown in Table 4, the total mixing time is 10 min.

Suspensions were prepared using a wall mixer DAC 600.2 VAC-P from the company Hauschild & Co.KG (Hamm, Germany). This model allows degassing during mixing by applying a vacuum. The range of rotational speed is 800–2350 rpm, and the maximum mixing capacity is 500 g.

#### 2.2.2. Lithography-Based Ceramic Manufacturing

**Figure 5:**
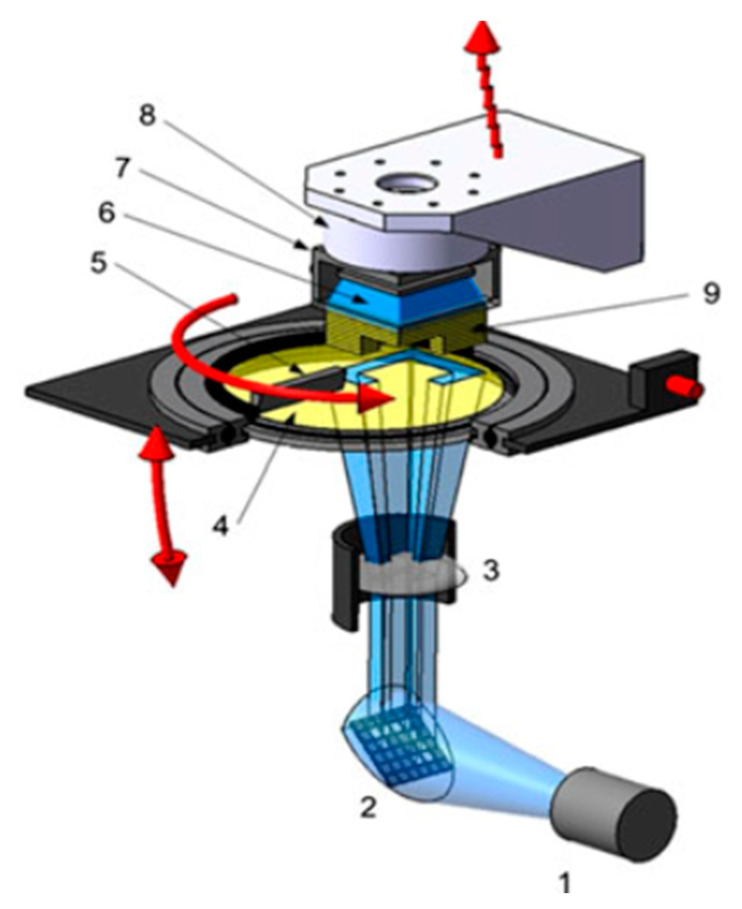
Schematic representation of an LCM machine: LED light source (1), DMD chip (2), optical lens (3), material vat (4), doctor blade (5), backwards exposure (6), building platform (7), load cell (8), part (9) [21]. Reproduced with permission from Passakorn Tesavibul; Ruth Felzmann; Simon Gruber; Robert Liska; Ian Thompson; Aldo R. Boccaccini; Jürgen Stampfl, Materials Letters; published by Elsevier, 2012.

LCM is a modified version of vat photo-polymerization—a modified version of the DLP(digital light processing)-based bottom-up vat photo-polymerization, to be exact. The machine used in this work was a prototype based on the patent WO2010045951 called Blueprinter 5 (built in-house). Figure 5 shows a schematic setup, and Table 5 shows certain specifications of this machine. Exposure happens through the transparent vat. The distinctiveness of this system is the rotating material vat, which enables coating by combination with the stationary doctor blade. Additionally, the vat can be tilted to ease the process of detaching the part from the vat surface. The main difference of LCM compared to conventional vat photo-polymerization is the use of ceramic suspensions. In this regard, LCM is a shaping method within the ceramic processing route [20]. If not stated otherwise directly in the respective paragraph, a layer height of 50 µm was used in every printing process described in this work.

#### 2.2.3. Debinding

**Figure 6:**
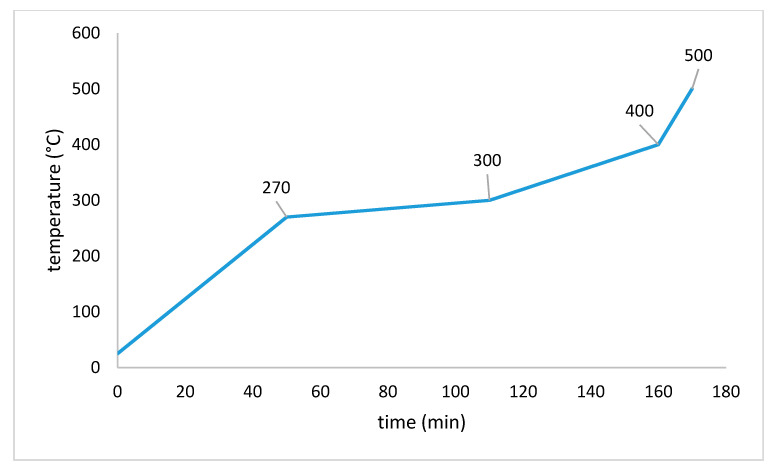
Debinding program E-MP/9.

The thermal debinding is executed in a HRF 7/22 convection oven from Carbolite Gero GmbH & Co.KG (Neuhausen, Germany). The temperature program (E-MP/9) is shown in Figure 6 [9]. For the debinding process, green bodies are embedded in a zirconia sand (Ø = 1 mm) from the company Powdercon (Biebbertal, Germany).

#### 2.2.4. Sintering

**Figure 7:**
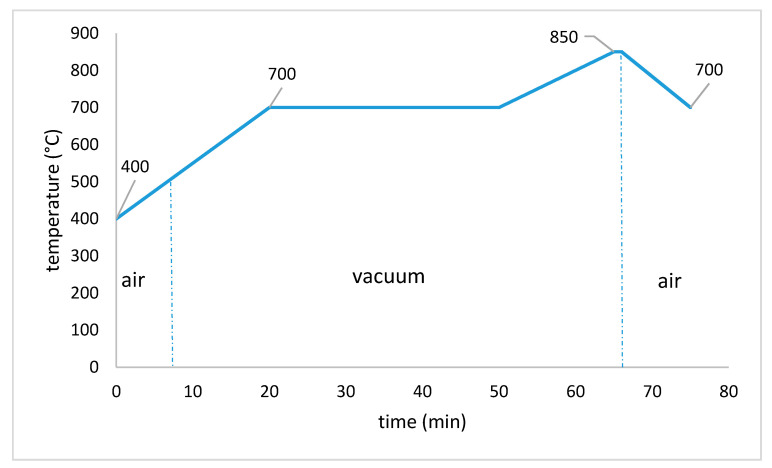
Sintering program RG_Lisi2_7_mod.6.

Sintering is performed in a Programat P700 dental oven from the company Ivoclar Vivadent AG (Schaan, Liechtenstein). This model is a small vacuum oven, which is appropriate for sintering lithium disilicate glass ceramics of the product group IPS e.max. The temperature program (RG_Lisi2_7_mod.6) is shown in Figure 7 [22].

### 2.3. Analyses

#### 2.3.1. Refractive Index

The refractive index of liquid substances was determined by using an Abbe refractometer. The temperature can be adjusted by a flow heater. In most cases in the literature, the n_D_^20^ is measured, meaning the refractive index at a temperature of 20 °C and at the wavelength of the D-line of a sodium vapor lamp (λ = 589 nm). In this work, the refractive index at a temperature of 23 °C (at λ = 589 nm) was measured to reproduce the conditions in the LCM machine.

#### 2.3.2. Exposure Tests

Exposure tests were conducted to determine the penetration depth D_p_ and the critical polymerization energy E_c_. D_p_ is the penetration depth of light into the measured material, at which the irradiance has decreased to 1/e (e being Euler’s constant). E_c_ is the exposure at which the material solidifies at least partially, meaning it is at the transition point from liquid to solid (gel point) [23].

They were performed by the following procedure: A plastic template with quadratic notches (edge length = 10 mm, depth = 3 mm) was put on a thin transparent polyethylene foil. Four notches were filled with the material to be measured. The filled template was put on the exposure area of the LCM machine, and each notch was subjected to a circular exposure field with a diameter of 6 mm. (The parameters of exposure were varied accordingly.) The (partially) cured objects were taken from the notches and their thickness was determined by using an outside micrometer. The average thickness of four specimens served as a value for the cure depth C_d_ at the chosen exposure (E_max_). Determining the border between solidified and residual material can be difficult, especially for specimens with a high refractive index difference, where the specimens tend to be gelled rather than cured. This leads to a relatively high uncertainty and an imperfect fitting of the linear models in some cases. Nevertheless, the data is essential for determining the behavior of the material during the LCM process.

Subsequently, the values for C_d_, depending on the exposure, were plotted in a half-logarithmical scale (Jacobs working curve). According to Equation (1), D_p_ is the slope of the resulting curve and E_c_ is the point of intersection with the x-axis. E_max_ is the maximum exposure (i.e., the exposure chosen for each measurement).

(1) $$C_{d}\;=D_{p}\;\ln\left(\frac{E_{\max}}{E_{c}}\right)$$

#### 2.3.3. Rheology

Viscosity measurements were performed with a MCR 300 oscillation rheometer from the company Anton Paar (Graz, Austria) at room temperature (25 °C) and by utilizing a CP50-1 cone-plate system with a diameter of 50 mm and a gap height of 100 µm. Samples consisted of all the constituents of the suspension formulation (solvent, monomer, dispersing agent, filler), except for photoinitiator and absorbing agent, to avoid rheological effects in case of undesired light exposure/polymerization.

#### 2.3.4. Thermal Analyses

Thermogravimetric analyses (TGA) were performed with a TGA Q500 from the company TA Instruments (New Castle, DE, USA). Cylindrical bodies with a height and diameter of 6 mm, produced by LCM, were measured. A heating rate of 1 K/min and a maximum temperature of 600 °C were chosen. Alumina crucibles with a low wall served as sample holders.

Thermo-mechanical analyses (TMA) were practical to determine the change of length of a specimen, depending on the temperature, and were conducted at a TMA Q400 of TA Instruments (New Castle, DE, USA). Cylindrical bodies with a height and diameter of 6 mm, produced by LCM, were measured. A heating rate of 1 K/min and a maximum temperature of 600 °C was chosen. Alumina crucibles with a high wall served as sample holders.

## 3. Results

### 3.1. Refractive Index

**Figure 8:**
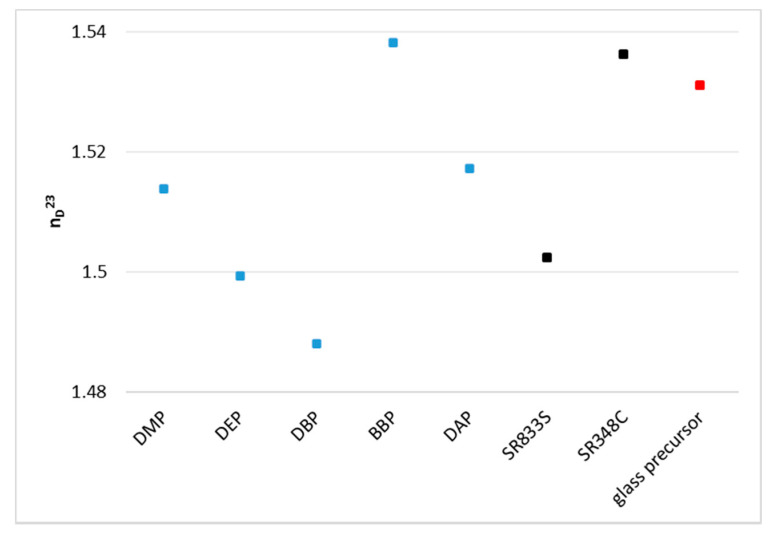
Comparison of the refractive indices of pure substances with the target value of the glass filler (value taken from literature [18]).

Halloran et al. showed that a small difference between the refractive index of the organic and the inorganic phase leads to a high precision of parts produced by AM based on photo-polymerization [12,13]. Therefore, it was one of the core considerations of this work to reduce this difference by choosing appropriate solvents. Accordingly, the refractive indices of the pure phthalates were first determined. The component BBP shows by far the highest refractive index of the solvents. Figure 8 compares these values to the refractive indices of the monomers and to the target value of the glass precursor. Only BBP shows a higher value than the glass precursor.

**Figure 9:**
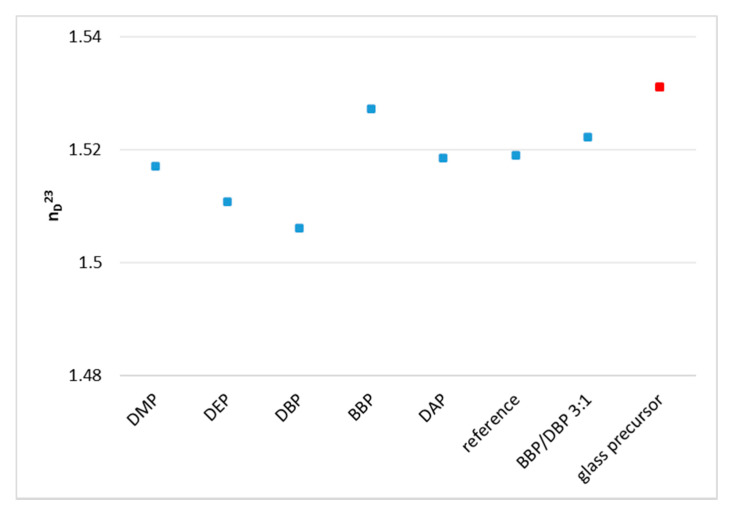
Refractive indices of the respective mixtures of each phthalate, the dispersing agent Solplus K500, and the monomer mixture of SR348C and SR833S.

As the next step, it was necessary to determine the refractive indices of the whole organic composition for each solvent. As could be anticipated, the composition with BBP came closest to the target value, as can be seen in Figure 9. The reference was a mixture without any solvent. In conclusion, from this group of solvents, the utilization of the component BBP is to be considered when a refractive index matching is desired.

### 3.2. Rheology

**Figure 10:**
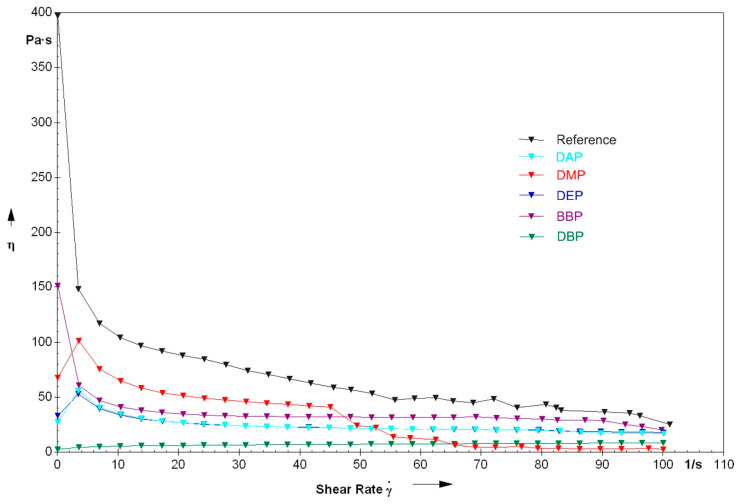
Viscosity in relation to shear rate for lithium disilicate suspensions prepared by using various phthalates.

The utilization of one of the mentioned phthalates significantly reduced the viscosity, especially in the range of low shear rates, as can be seen in Figure 10. Emphasis is to be put on the composition with DBP, which showed the lowest viscosity in the range from 0–65/s and was different from the other compositions in that it showed very low viscosity at low shear rates. This resulted in the composition being flowable, but not stable against sedimentation.

**Figure 11:**
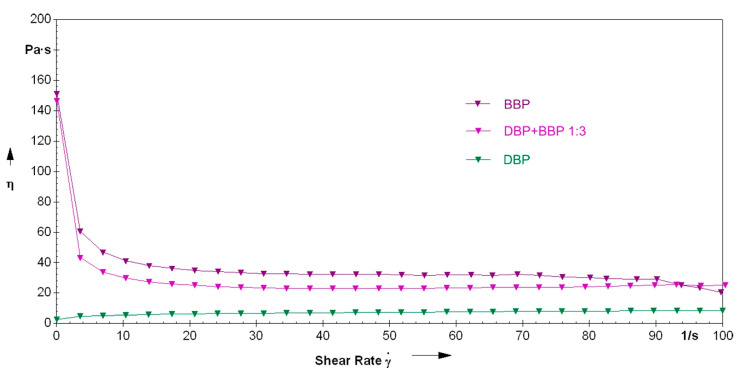
Direct comparison of the rheological properties of the formulations with either BBP, DBP, or a 3:1 mixture of the two.

Figure 11 shows the measurements for compositions with BBP and DBP, together with a composition with a 3:1 mixture of the two solvents. On the one hand, the flowability of the DBP suspension is advantageous, because it allows an uncomplicated processing. On the other hand, the rise of viscosity at low shear rates is advantageous, because it increases resistance to sedimentation. The mixture of those two phthalates makes a good compromise. On the one hand, the viscosity, compared to BBP, is decreased, and a certain flowability is present. On the other hand, the viscosity is high enough to enable stability against sedimentation, especially because of the increase of viscosity at low shear rates.

### 3.3. Debinding

**Figure 12:**
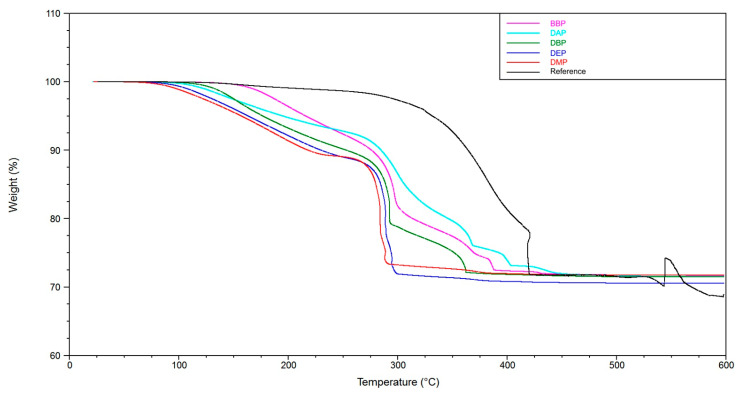
Thermo-gravimetric analysis of green bodies produced by LCM using suspensions with varying solvents.

Figure 12 shows the TGA results. In the case of the phthalate suspensions, we can see a two-step process, with the first step being the evaporation of solvents in a temperature range from 75 to 250 °C. The weight loss for the reference, about 1% in this temperature range, is negligible. The second step is the degradation of the polymer network in a temperature range from 250 to 450 °C. Compared to the reference, we can see that this step is completed at lower temperatures for the phthalate suspensions when the solid loading of 72.5 wt% is reached (DMP: 290 °C, DEP: 300 °C, DBP: 360 °C, BBP: 390 °C, DAP: 410 °C, reference: 425 °C).

**Figure 13:**
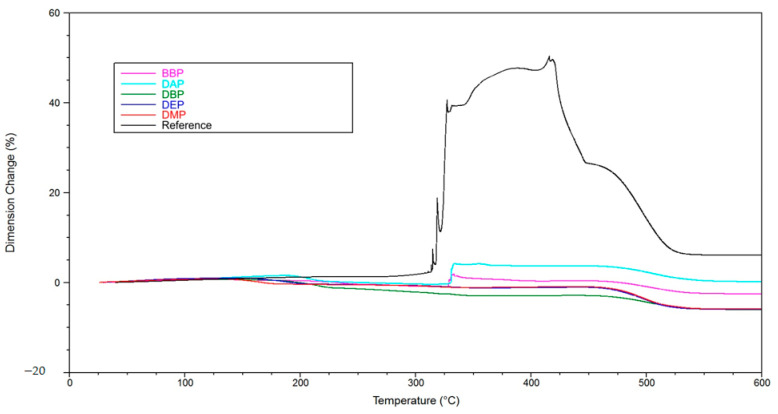
Thermomechanical analysis of green bodies produced by LCM using different phthalate suspensions.

In TMA measurements (Figure 13), a positive influence of the phthalates can be recognized as well. The maximum expansion for the various phthalate suspensions is under 10%, while the reference shows a maximum expansion of 50%. Together with several spikes showing rapid expansion, this means complete destruction of the part, as can be seen in Figure 15a.

**Figure 14:**
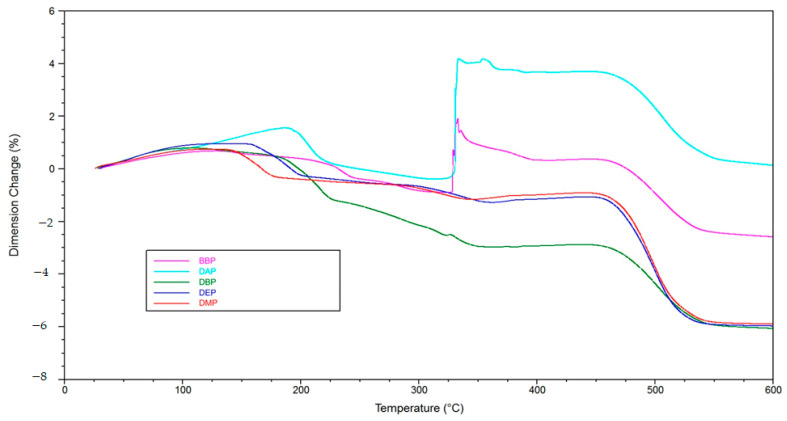
Detailed depiction of the thermomechanical analysis from Figure 13.

**Figure 15:**
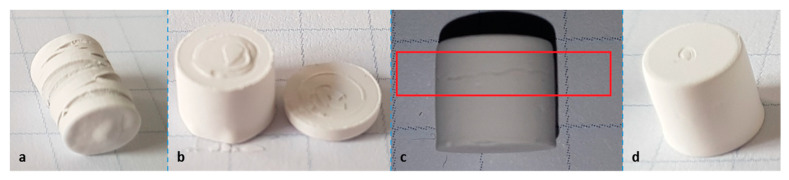
Specimens after the thermomechanical analysis from Figure 13; (**a**) reference, (**b**) DAP, (**c**) BBP, (**d**) DBP.

In Figure 14, the reference curve is removed, and a detailed depiction of the phthalate suspensions is shown. It allows one to see that in the case of DAP and BBP, rapid expansion occurs at temperatures between 325 and 350 °C. As mentioned, this means crack extension, as can be verified in Figure 15b,c. The DBP sample is shown as an opposite example. In this case, no crack extension could be observed (Figure 15d).

As mentioned previously, the component BBP is necessary when a refractive index matching is desired. Should crack extension be unavoidable during debinding (at considerably low heating rates of 1 K/min), this component needs to be discarded. To allow at least a partial utilization of BBP, it was attempted to specifically mix in a certain amount of DBP, since it showed the lowest expansion during TMA measurements, and additionally, had a positive influence on the rheological properties (increase of flowability).

**Figure 16:**
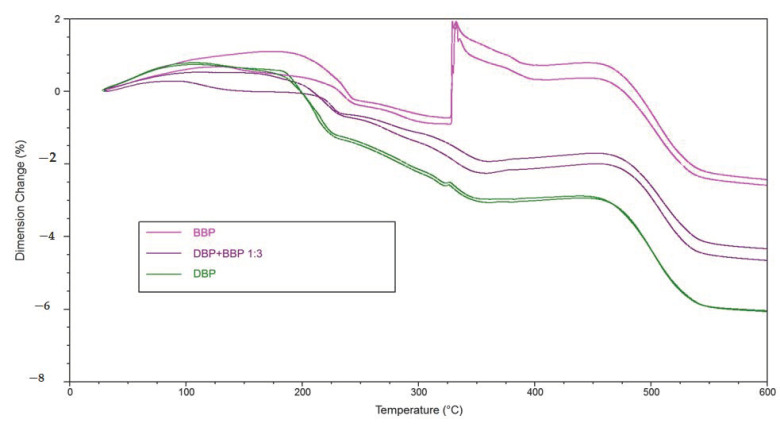
Direct comparison of the thermomechanical measurements (two each) of green bodies, produced by LCM with BBP, DBP, and 3:1-mixture of both, respectively.

Figure 16 shows the direct comparison of the TMA measurements of green bodies produced by LCM using suspensions with either BBP, DBP, or a 3:1 mixture of the two solvents. To make sure the results are reproducible, two single measurements were performed in each case. For the mixture of BBP/DBP, there is no crack extension observable. Even the small peak at 325 °C, in the case of DBP, could be avoided. A possible explanation is that DBP exits the green body slightly earlier (bp. 340 °C) than BBP (bp. 370 °C) and leaves open porosity, which facilitates the exit of BBP and of degradation products of the polymer network later.

### 3.4. Exposure Tests

**Figure 17:**
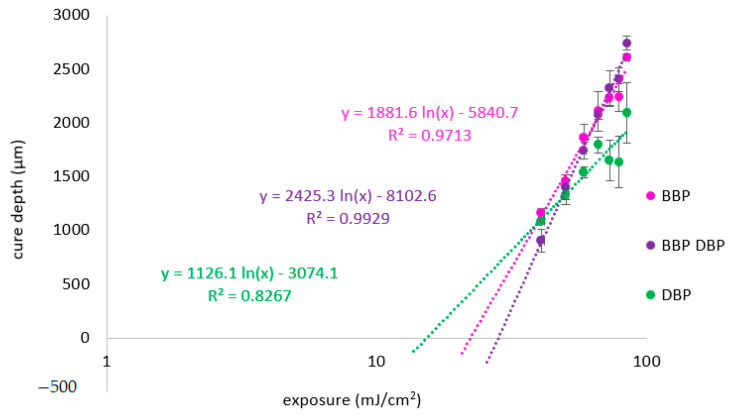
Jacobs working curve for lithium disilicate suspensions prepared by using various phthalates and no absorbing agent.

To characterize the behavior of the different phthalate suspensions during the exposure in the AM process, exposure tests were performed. Figure 17 shows the Jacobs working curve for the especially relevant suspensions with BBP, DBP, and a 3:1 mixture of the two. Table 5 lists the parameters calculated from the working curves.

According to theory, D_p_ should increase with decreasing difference of refractive index between organic mixture and filler of the suspension. The results for DBP and BBP fit well to this expectation (Table 6). The refractive index difference is comparatively high for the DBP suspension, therefore D_p_ is low. The refractive index difference is low for the BBP suspension, therefore D_p_ is high. Opposingly, it is surprising that the highest value is achieved for the mixture of the two phthalates. Previously, we have seen that the refractive index matching is best for the BBP suspension. Therefore, the highest value was expected for the BBP sample.

Two explanations are possible. On the one hand, the refractive index of the material rises during polymerization. Should this rise in refractive index lead to a better matching, this could explain the higher D_p_. On the other hand, the target value of the glass filler is a literature value. In the literature, it is already noted that the refractive index (depending on the batch) can vary between 1.528 and 1.534. Consequently, the exact target value for this batch is not known and should be determined experimentally.

**Figure 18:**
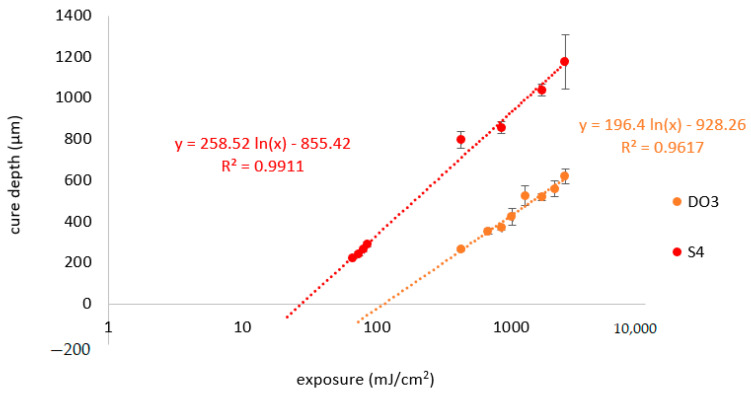
Jacobs working curve for lithium disilicate suspensions prepared by using a 3:1-mixture of BBP and DBP and either Sudan IV or Disperse Orange 3 as absorber.

The high penetration depths of the phthalate suspensions lead to the necessity for high concentrations of absorbing agents to effectively decrease D_p_. In this work, two substances were examined for this purpose: Disperse Orange 3 (DO3) and Sudan IV (S4). Figure 18 shows the Jacobs working curves for the suspensions with a 3:1 mixture of BBP/DBP, with a concentration of 0.03% with regards to the total mass of the suspension for DO3 and S4, respectively.

Both absorbing agents lead to similar values for D_p_, as can be seen in Table 6. It is noticeable that in the case of DO3, E_c_ is increased by a factor of four, compared to the version without absorbing agent (Table 7). In the case of S4, E_c_ remains nearly unchanged.

**Figure 19:**
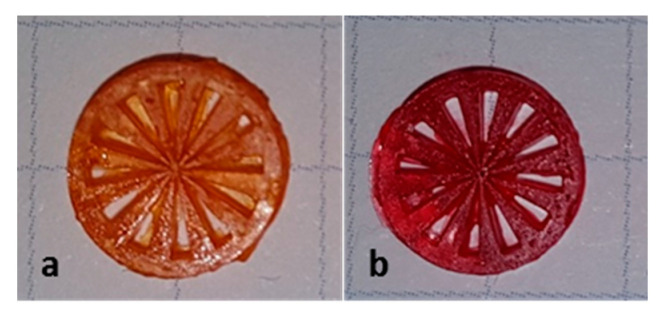
Green bodies of Siemens stars produced by LCM, using Disperse Orange 3 (**a**) and Sudan IV (**b**).

These perceptions are relevant for the exposure time in the AM process. Figure 19 shows two siemens stars, produced by LCM using a suspension with a 3:1 mixture of BBP/DBP with either DO3 (a) or S4 (b) in the same concentration (0.03%). In the case of DO3, an exposure time of 10 s was needed. In the case of S4, an exposure time of 1 s was needed. In both cases, an exposure of 85 mW/cm^2^ was applied.

## 4. Discussion

### 4.1. Refractive Index

Using phthalates, the refractive index of the organic fraction can be increased significantly and therefore matched closely to the refractive index of the inorganic phase. By choosing a 3:1 mixture of BBP/DBP, the difference between organic and inorganic phase is Δn = 0.0089.

Some issues persist with regards to a perfect refractive index matching. Firstly, the target value is a literature value. The refractive index of the filler varies according to the producer, with a relatively big margin (*n* = 1.531 ± 0.003). The experimental determination of the refractive index of the glass powder batch at hand should give a more exact and reliable value. Secondly, the refractive indices of all the components of the formulation should be determined at processing temperature and processing wavelength to reach the target value precisely. The presented solvents are an appropriate tool for that.

### 4.2. Debinding

The TGA and TMA experiments clearly show two things. On the one hand, the utilization of a solvent enables a debinding procedure without crack extension (see reference without solvent). On the other hand, the choice of solvent is a deciding factor for the speed that can be applied to this procedure. At a heating rate of 1 K/min, the application of BBP and DAP leads to crack extension. In the case of DMP, DEP, and DBP, this heating rate could be used without crack extension. An additional observation is that by adding a component that is easily debindable (in this case DBP), the crack extension can effectively be avoided. Accordingly, a 3:1 mixture of BBP/DBP leads to a successful debinding procedure at the same heating rate.

A possible explanation is that the DBP molecules, due to their chemical constitution, possess a weaker intermolecular interaction with the other components inside the green body, for example BBP, and exit the green body at lower temperatures (TGA, Figure 12). An interesting subject for future work would be to determine the intermolecular interactions by quantum mechanical calculations. We estimate that, consequently, more open porosity is present in the green body that facilitates the exit of polymer degradation products at a later stage of the procedure. If both mechanisms happen chronologically separated, a successful debinding is to be expected. In the case of BBP, the exit of the solvent molecules happens at a higher temperature/later in time, which is the reason why, presumably, not enough open porosity is present when the degradation of the polymer network starts. Consequently, a higher pressure builds up inside the green body, which eventually leads to fracture of the sample, as shown in the TMA measurements (Figure 13 and Figure 14). Therefore, it is necessary to choose an easily debindable component that establishes enough open porosity early on, before the degradation of the polymer network begins.

### 4.3. Rheology

Likewise, regarding rheology, the choice of the solvent is of major significance. Accordingly, the viscosity of a suspension prepared without a solvent is at 100–75 Pa·s for a shear rate range of 15–45 s^−1^. Opposingly, the viscosity of the suspension prepared with the 3:1 mixture of BBP/DBP is at 30 Pa·s for the same shear rate range.

An explanation for the different rheological behavior of the DBP suspension could be the already mentioned weaker intermolecular forces. Due to the lipophile character of DBP (compared to, for example, DMP), the non-covalent network structure of the suspension is not as pronounced. This network structure seems to be significant for the buildup of stability.

### 4.4. Absorbing Agent

The results of the exposure tests could identify a deciding disadvantage of DO3: at a concentration that is necessary to reduce the penetration depth to an appropriate extent, the critical polymerization energy is increased by a factor of four. This shows an inhibition of the polymerization by the mono azo dye. We assume that the reason for this is the nitro function on the phenyl ring, which acts as a radical quencher by mesomeric effect. With Sudan IV, an alternative option was presented that can reduce D_p_ effectively without increasing E_c_. With this alternative, the exposure time could be reduced from 10 s to 1 s.

Conclusively, it was shown that by changing the solvent, many important properties of a photo-reactive suspension for the additive manufacturing of glass ceramics can be influenced, and by combination, those properties can be adapted according to requirements.

Future investigations should focus on other groups of solvents to improve the refractive index matching even further, and to see if shown benefits can be reproduced with other chemical substances. Additionally, it should be investigated if these considerations regarding the formulation of a photo-reactive suspension are also valid for other types of glasses or glass ceramics, and how these changes affect the final properties of the ceramics, like translucency/transparency or mechanical strength.

## Figures and Tables

**Table 1:** Typical formulation for a photo-reactive suspension used in lithography-based ceramic manufacturing (LCM).

Component	Mass Fraction (%)
Solvent	10–20
Monomer	10–20
Photoinitiator	<0.5
Dispersant	<5
Absorber	<0.1
Ceramic filler	60–80

**Table 2:** Chemical composition of the cast blanks.

Component	Mass Fraction (%)
SiO_2_	57–80
Li_2_O	11–19
K_2_O	0–13
P_2_O_5_	0–11
ZrO_2_	0–8
ZnO	0–8
Al_2_O_3_	0–5
MgO	0–5
Coloring Oxides	0–8

**Table 3:** Composition of the photo-reactive suspensions.

Component	Mass Fraction (%)
Solvent	11.61
Monomers	14.53
Dispersant	1.08
Photoinitiator	0.20
Absorber	0.03
Glass powder	72.55

**Table 4:** Program for preparing and degassing glass suspensions.

Program Step	Mixing Duration (min)	Rotational Speed (rpm)	Pressure (mbar)
1	2	2350	1000
2	3	800	500
3	5	800	100

**Table 5:** Specifications of the LCM prototype.

Specification	BP5
Exposure system	DLP
Emission wavelength (nm)	460
Resolution (pixels)	1920 × 1200
Pixel size (µm)	40
Vat diameter (mm)	150
Building volume (wxdxh) (mm)	77 × 43 × 110

**Table 6:** Penetration depth and critical polymerization energy, calculated from Figure 17.

Suspension	D_p_ (µm)	E_c_ (mJ/cm^2^)
DBP	1126	15
BBP	1882	22
BBP/DBP 3:1	2425	28

**Table 7:** Penetration depth and critical polymerization energy, calculated from Figure 18.

Suspension	D_p_ [µm]	E_c_ [mJ/cm^2^]
Disperse Orange 3	196	113
Sudan IV	259	27

## Data Availability

The data presented in this study are available on request from the corresponding author. The data are not publicly available due to a retention period.
